# Concurrent presentation of an intraductal tubulopapillary neoplasm and intraductal papillary mucinous neoplasm in the branch duct of the pancreas, with a superior mesenteric artery aneurysm: a case report

**DOI:** 10.1186/s12957-018-1391-9

**Published:** 2018-04-24

**Authors:** Kenta Inomata, Minoru Kitago, Hideaki Obara, Yoko Fujii-Nishimura, Masahiro Shinoda, Hiroshi Yagi, Yuta Abe, Taizo Hibi, Kentaro Matsubara, Go Oshima, Yasuhito Sekimoto, Masazumi Inoue, Osamu Itano, Michiie Sakamoto, Yuko Kitagawa

**Affiliations:** 1 Department of Surgery, Keio University School of Medicine, 35 Shinanomachi, Shinjuku-ku, Tokyo, 160-8582 Japan; 2 Department of Pathology, Keio University School of Medicine, Tokyo, Japan; 3 Department of Transplantation/Pediatric Surgery, Kumamoto University Graduate School of Medical Sciences, Kumamoto, Japan; 4 Department of Hepato-Biliary-Pancreatic and Gastrointestinal Surgery, International University of Health and Welfare School of Medicine, Chiba, Japan

**Keywords:** Pancreatic neoplasm, Pancreatic cyst, Pancreaticoduodenectomy

## Abstract

**Background:**

Since the concept of intraductal tubulopapillary neoplasm (ITPN) was introduced in the current World Health Organization classification of tumors, the number of reports of ITPN occurrence has increased gradually. However, ITPN is usually located in the main pancreatic duct, with few reports of a branch duct ITPN. As a result, imaging protocols for the diagnosis of a branch duct ITPN have not been established.

**Case presentation:**

We report a case of a concurrent presentation of a branch duct ITPN and intraductal papillary mucinous neoplasm (IPMN) in the head of the pancreas, with a superior mesenteric artery (SMA) aneurysm. Initially, the cystic masses in the pancreatic head were diagnosed as branch duct IPMNs, with treatment consisting of a pylorus-preserving pancreaticoduodenectomy, in combination with an aneurysmectomy performed for treatment of the SMA aneurysm. Pathological examination confirmed these cysts were a combination of branch-type ITPN and IPMN. The patient recovered from the treatment without complication, with no evidence of recurrence over a period of 34 months post-surgery.

**Conclusion:**

This case report of a synchronous presentation of an ITPN and IPMN indicates the difficulty in differentiating these two types of neoplasms in the branch duct of the pancreatic head.

## Background

Cystic lesions in the pancreas have been identified with increasing frequency in recent years due to the development and advancement of imaging technology. Although some of these cystic tumors, such as intraductal papillary mucinous neoplasms (IPMNs), have a potential for malignancy that cannot be ignored, considering the risks associated with pancreatic resection, surgical treatment must be considered with caution. Therefore, accurate imaging is clinically important to establish a diagnosis and indication for surgery.

Intraductal tubulopapillary neoplasms (ITPNs) were introduced as a new type of pancreatic tumor by Yamaguchi et al. in 2009 [1] and subsequently included as an independent entity in the current World Health Organization classification of tumors in 2010. ITPNs show a predominantly tubular growth pattern, with a papillary component and absence of intracytoplasmic mucin [2]. These features differentiate ITPN from intraductal papillary mucinous neoplasm (IPMNs) and pancreatic intra-epithelial neoplasia. Although the clinical course of ITPN has been reported to be indolent, with a better prognosis than for invasive ductal adenocarcinoma [1, 3, 4], an ITPN may have invasive features and can metastasize to lymph nodes or the liver [1]. While the number of case reports on ITPNs is limited, the report of an ITPN in a branch duct of the pancreas is even rarer as these tumors are usually located in the main pancreatic duct. Therefore, image findings for ITPN diagnosis and surgical indication remain unclear.

An aneurysm of the superior mesenteric artery (SMA) is also a rare occurrence, accounting for only 6.9% of all visceral artery aneurysms [5], and carries the risk for embolism or rupture, with subsequent mesenteric ischemia and massive hemorrhage [6]. Surgical intervention is recommended to prevent these fatal complications [5, 6]. Although endovascular treatment can be effective for the treatment of a SMA aneurysms, open surgery, including aneurysmectomy and pancreaticoduodenectomy, may be required depending on the characteristics of the aneurysm [5–7]. In this case report, we describe the presentation and clinical diagnosis of a patient with a rare occurrence of a concurrent presentation of branch duct ITPN and IPMN in the pancreatic head, with a SMA aneurysm.

## Case presentation

A 55-year-old woman presented to a local hospital complaining of epigastralgia and anemia. Endoscopic examination of the upper gastrointestinal tract revealed gastritis, with pancreatic masses observed on abdominal ultrasonography. Computed tomography (CT) imaging further revealed an aneurysm of the SMA. Based on these findings, the patient was admitted to our hospital for further investigation and treatment.

**Fig. 1:**
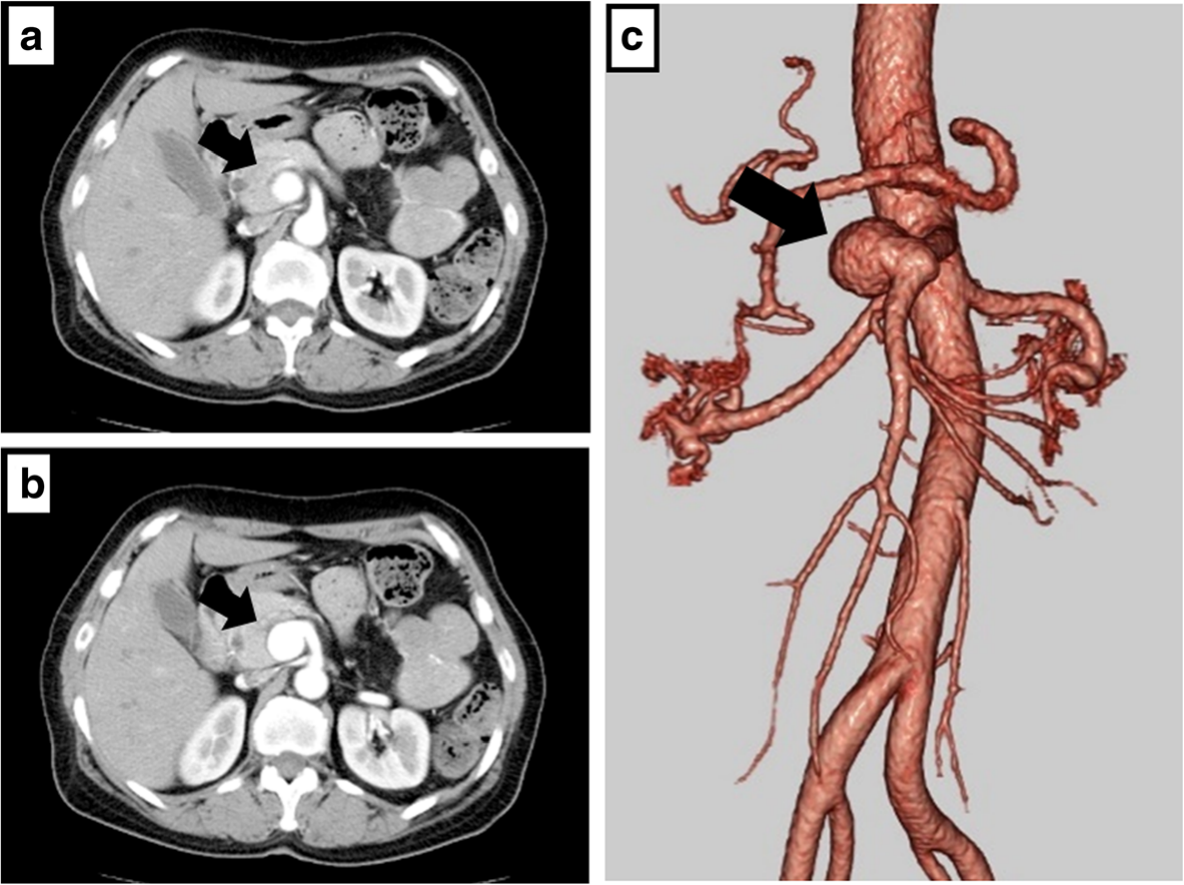
Axial contrast-enhanced computed tomography (**a**, **b**) and 3-dimensional image reconstruction (**c**) showing a 2.4-cm saccular aneurysm of the superior mesenteric artery (arrow)

**Fig. 2:**
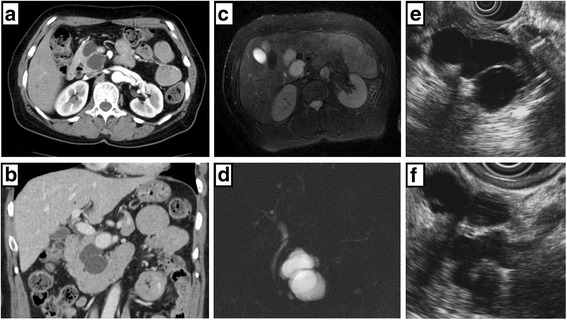
Axial (**a**) and coronal (**b**) contrast-enhanced computed tomography showing a cystic mass in the pancreatic head. Fat-suppressed T2-weighted magnetic resonance imaging (**c**) and magnetic resonance cholangiopancreatography (**d**), showing a 50 × 33-mm cystic mass, without dilation of the main pancreatic duct. Endoscopic ultrasound image, showing a 50-mm hypoechoic area in the pancreatic head (**e**). Mural nodules and dilation of the main pancreatic duct upstream, in the distal pancreas, were not observed (**f**)

Her medical history included hypertension, cerebral hemorrhage, and panic disorder, with no indication of hyperglycemia. Findings on physical examination were unremarkable and laboratory data, including tumor markers, were within normal limits. Dynamic CT imaging revealed a saccular SMA aneurysm, with a 2.4-cm diameter, located 2 cm distal to the origin of the SMA (Fig. 1a–c), as well as a multiloculated cystic mass in the pancreatic head, with no mural nodule on enhanced CT imaging (Fig. 2a, b). Magnetic resonance (MR) imaging confirmed the cystic masses in the pancreatic head (Fig. 2c), but with no indication of dilation of the main pancreatic duct on MR cholangiopancreatography (Fig. 2d). Endoscopic ultrasonography revealed a 5-cm hypoechoic area in the pancreatic head, but with no evidence of a mural nodule and dilation of the main pancreatic duct upstream, in the distal pancreas (Fig. 2e, f). Based on these findings, a preoperative clinical diagnosis of branch duct IPMN, with a concurrent SMA aneurysm, was made, with a serous cystic neoplasm and pseudocyst included in the differential diagnosis.

**Fig. 3:**
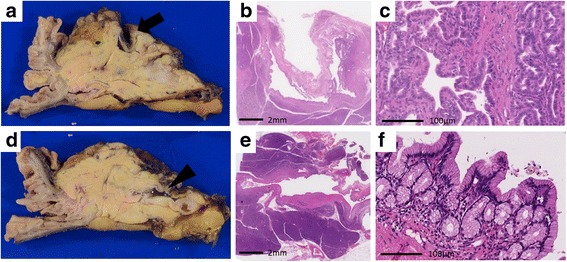
Macroscopic assessment revealed one of the two cystic masses to be an intraductal tubulopapillary neoplasm (ITPN; arrow) and the other an intraductal papillary mucinous neoplasm (IPMN; arrowhead) (**a**, **d**). Features of the ITPN included neoplastic cells with enlarged nuclei, with the eosinophilic cytoplasm contains little mucus (**b**, **c**). In contrast, the IPMN showed neoplastic cells, with obvious mucin in well-developed tubules (**e**, **f**)

**Fig. 4:**
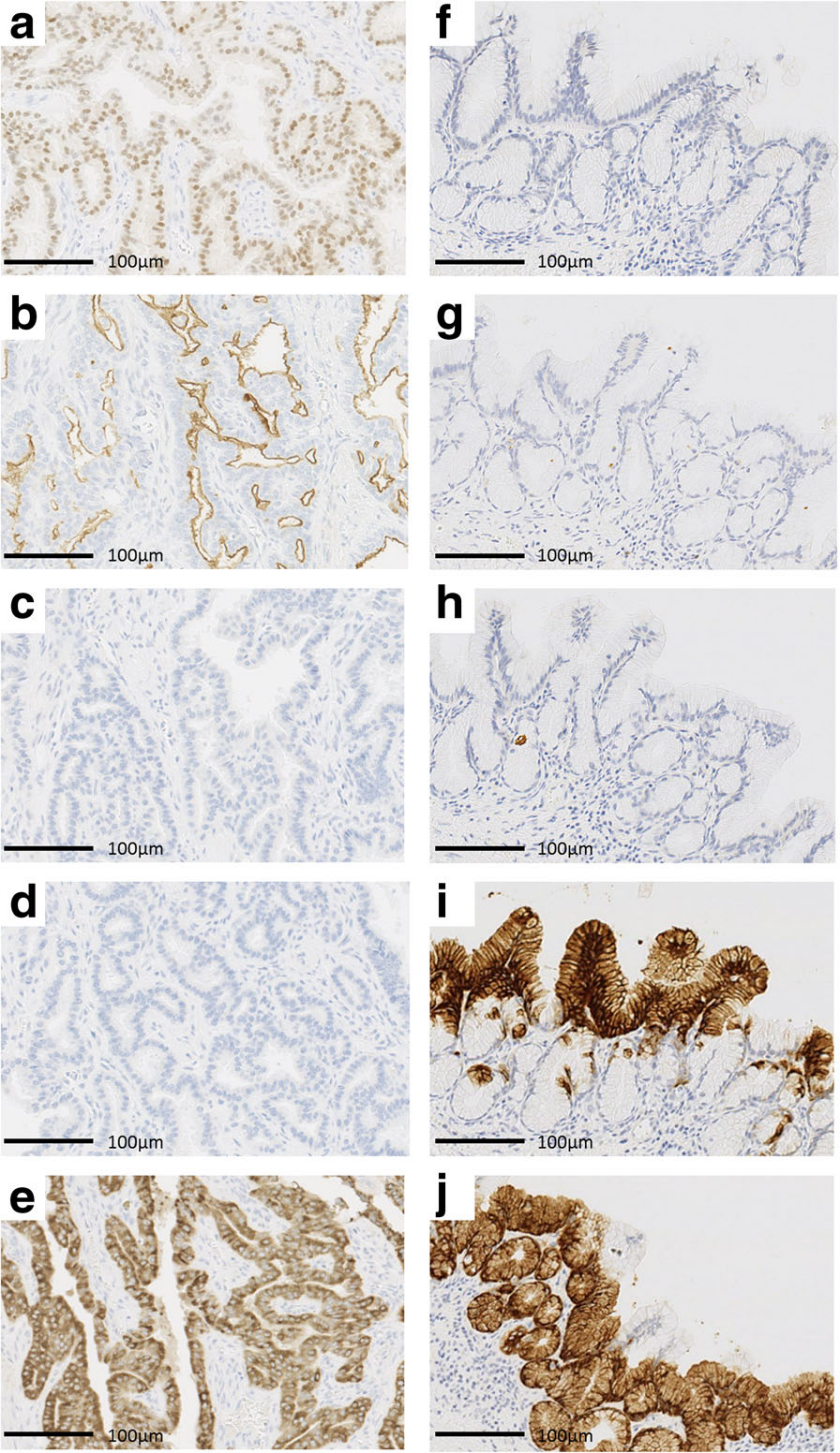
Representative micrographs of the intraductal tubulopapillary neoplasm (ITPN; **a**–**e**) and the intraductal papillary mucinous neoplasm (IPMN; **f**–**j**) specimens, immunostained for CDX-2 (**a**, **f**), MUC-1 (**b**, **g**), MUC-2 (**c**, **h**), MUC-5 AC (**d**, **i**), and MUC-6 (**e**, **j**). ITPN stained positive for CDX-2 (**a**), MUC-1 (**b**), and MUC-6 (**e**), and negative for MUC-2 (**c**) and MUC-5 AC (**d**). IPMN stained positive for MUC-5 AC (**i**) and MUC-6 (**j**), and negative for CDX-2 (**f**), MUC-1 (**g**), and MUC-2 (**h**)

After obtaining informed consent, we simultaneously performed a pylorus-preserving pancreatoduodenectomy and SMA aneurysmectomy. Macroscopic examination revealed the presence of two cystic masses in the pancreatic head. Neoplastic cells were identified in one of these masses, characterized by enlarged nuclei and eosinophilic cytoplasm containing little mucus (Fig. 3a–c). The other cystic mass showed a proliferation of low-papillary columnar cells, with intracellular mucus (Fig. 3d–f). Immunohistochemical examination revealed positive staining of the mucin-poor region of the first cystic mass for MUC-1, MUC-6, and CDX-2, but negative for MUC-2 and MUC-5 AC (Fig. 4a–e), with an approximate Ki-67 index in this region of 1%. Based on these findings, a diagnosis of ITPN was confirmed. For the second cystic mass, the mucin-rich region stained positive for MUC-5 AC and MUC-6, but negative for CDX-2, MUC-1, and MUC-2 (Fig. 4f–j), with an approximate Ki-67 index in this region of < 1%. Based on these results, this mass was diagnosed as an intraductal papillary mucinous adenoma with moderate atypia. The final histologic diagnosis for the first mass was an ITPN, with high-grade dysplasia, and branch duct-type IPMN, with moderate atypia, for the second mass. There was no evidence of malignancy or lymph node metastasis.

The patient’s postoperative course was uneventful and her length of hospital stay was 35 days. She has remained symptom-free since treatment, with no recurrence of the neoplasm over the 34-month follow-up.

## Discussion and conclusions

It has been reported that ITPN accounts for only 0.9% of all pancreatic exocrine tumors and 3% of all pancreatic intraductal neoplasms [1]. Although the pathological definition of ITPNs has been established, because of their rarity, their clinical and imaging features for diagnosis have not been clearly defined. Several studies have suggested that preoperative diagnosis of intraductal tubular neoplasms to be difficult [8–11]. With specific regard to the diagnosis of an ITPN, Oh et al. [11] and Ishigami et al. [10] suggested that upstream dilation of the main pancreatic duct may be a specific finding to differentiate an ITPN from an IPMN. Ishigami et al. reported three cases of intraductal tubular neoplasms that were hypovascular tumors without downstream pancreatic duct dilation on CT and MR imaging [10]. Furthermore, Motosugi et al. described the “2-tone duct sign” and “cork-of-wine-bottle sign” as characteristic findings of ITPNs on diagnostic imaging [12]. However, these features are specific to neoplasms of the main pancreatic duct, which is the most common site for pancreatic ITPNs, with reports of branch duct ITPNs being very limited. Yoshida et al. reported an ITPN of the pancreatic branch duct which presented as a round, well-circumscribed, weakly enhanced hypovascular mass [13]. In our case, the cystic masses did not demonstrate typical findings of a main duct ITPN, with their features, in fact, being more consistent of a branch duct ITPN or IPMN. Although endoscopic retrograde cholangiopancreatography or transpapillary biopsy was not performed in our case as the IPMN was located in a branch duct, some reports have demonstrated that transpapillary biopsy or brush may be useful for preoperative diagnosis [2, 8].

Initially, we had diagnosed the two cystic masses as IPMNs. According to the international consensus guidelines for the management of IPMNs (2012), immediate resection is not indicated (although the decision should be made on a case-by-case basis) for branch duct IPMNs with a diameter > 3 cm and without observable mural nodules [14]. In the present case, the cyst was approximately 5 cm in diameter and there were no other risk factors, such as mural nodule or positive cytology. We considered a pancreaticoduodenectomy to be an appropriate treatment based on the characteristics of the IPMNs, while also taking into consideration the patient’s symptoms, surgical risk, the patient’s relatively young age, and absence of comorbidities, as well as the presence of a SMA aneurysm.

Histologic examination of the two cystic tumors identified on preoperative imaging confirmed the presence of two types of neoplasms, namely an ITPN and an IPMN. Yamaguchi et al. described the pathological characteristics of an ITPN as follows: (1) a solid nodular tumor obstructing dilated ducts on macroscopic examination, (2) no visible secretion of mucin, (3) tubulopapillary growth, (4) uniform high-grade atypia throughout the neoplasm, (5) an easily recognizable necrotic foci, (6) ductal differentiation, and (7) absence of acinar differentiation [1]. Immunohistochemical staining is useful to confirm the ITPN, with MUC-1, MUC-6, and CDX-2 tend to be expressed, with negative staining for MUC-2 and MUC-5 AC [1]. In our case, the neoplasm presented a tubulopapillary growth pattern, without visible mucin secretion and positive immunohistochemical staining for MUC-1, MUC-6, and CDX-2, and negative results for MUC-2 and MUC-5 AC confirmed the diagnosis. For the second cyst, positive results for MUC-5 AC and MUC-6, and negative results for MUC-1, MUC-2, and CDX-2 were consistent with a diagnosis of IPMN [15].

In general, the prognosis for ITPNs is considered to be much better than the poor prognosis for pancreatic ductal adenocarcinoma [1, 16]. The largest published case series to date reported on the outcomes of 10 cases of ITPNs, three of which had stromal invasion extending to the veins, bile ducts, and duodenum [1]. Among these cases, one patient died of multiple liver metastases from the neoplasm at 7 months after primary resection. In the remaining cases, there was no evidence of metastasis to the regional lymph nodes, with all patients being still alive up to 7 years after diagnosis. It has been suggested that the Ki-67 labeling index may be predictive of the prognosis [1]. In our case, the patient was still alive 34 months after diagnosis and treatment, with no evidence of recurrence.

With respect to SMA aneurysms, surgical interventions including pancreatic resection and stent-grafting are recommended treatments due to the risk of rupture [5]. In our case, as the aneurysm was relatively close to the origin of the SMA and first jejunal artery and had a wide neck, we selected to proceed with open surgery over stent-grafting or simple coil embolization. Pancreaticoduodenectomy was theoretically a suitable procedure as the aneurysm was located just behind the pancreas, and fortunately, the cystic tumor in the pancreatic head could be resected at the same time.

We identified 44 articles published in English addressing ITPN in PubMed using the keywords “ITPN” and “intraductal tubulopapillary neoplasm,” as well as four Japanese articles from other sources. Eight articles addressing ITPN in the bile duct were excluded, while another 10 articles were excluded because they did not provide detailed patient data. Therefore, we extracted comprehensive data on 51 cases of ITPN from 26 articles [1–4, 12, 13, 17–36]. Among these cases, only six cases (11.8%) arose from the branch duct. In addition, there was no report mentioning an association between ITPN and IPMN.

Therefore, our case of a concurrent branch duct ITPN and IPMN in the pancreatic head, with a SMA aneurysm, is a very rare occurrence, with an association between an ITPN and IPMN not previously having been reported. Our case underlines the difficulty in differentiating these two types of neoplasm pre-operatively. Therefore, an ITPN should be included in the differential diagnosis, even when a cystic mass suggestive on an IPMN is observed.

## Abbreviations

CT: Computed tomography
IPMN: Intraductal papillary mucinous neoplasm
ITPN: Intraductal tubulopapillary neoplasm
MR: Magnetic resonance
SMA: Superior mesenteric artery
